# Mechanisms of Rhizoma Coptidis against type 2 diabetes mellitus explored by network pharmacology combined with molecular docking and experimental validation

**DOI:** 10.1038/s41598-021-00293-8

**Published:** 2021-10-21

**Authors:** Wenrong An, Yanqin Huang, Shouqiang Chen, Tao Teng, Yingning Shi, Zhenhai Sun, Yunsheng Xu

**Affiliations:** 1 First Clinical Medical College, Shandong University of Traditional Chinese Medicine, Jinan, 250355 China; 2 Department of Endocrinology, Affiliated Hospital of Shandong University of Traditional Chinese Medicine, Jinan, 250014 China; 3 Department of Endocrinology, Second Affiliated Hospital of Shandong University of Traditional Chinese Medicine, No. 1 Jingba Road, Jinan, 250001 China

**Keywords:** Computational biology and bioinformatics, Endocrinology

## Abstract

This study systematically explored the underlying mechanism of Rhizoma Coptidis against type 2 diabetes mellitus (T2DM) by using network pharmacology and molecular docking and experimental validation. We retrieved and screened active compounds of Rhizoma Coptidis and corresponding T2DM-related targets across multiple databases. PPI networks of the genes were constructed using STRING, and the core targets were screened via topological analysis. GO and KEGG enrichment analyses were performed by using DAVID. Finally, molecular docking and experimental studies were performed after bioinformatic analysis for verification. There were 14 active compounds and 19 core targets of Rhizoma Coptidis-T2DM, of which quercetin was identified as the main compound and IL6, VEGFA and TNF were the most significant core targets. GO and KEGG enrichment analyses showed that Rhizoma Coptidis ameliorated T2DM by regulating multiple biological processes and pathways. Docking studies indicated that IL6, VEGFA and TNF could stably bind with all active compounds of Rhizoma Coptidis. The results of our experiments revealed that Rhizoma Coptidis could inhibit the expression of IL6 and TNFα and enhance islet cell viability. This study suggests anti-inflammatory therapeutic effects of Rhizoma Coptidis on T2DM, thereby providing a scientific basis and new insight for further research on the antidiabetic effect of Rhizoma Coptidis.

## Introduction

Type 2 diabetes mellitus (T2DM) is a metabolic disease triggered by insulin resistance or impaired insulin secretion and characterized by hyperglycaemia^1,2^. T2DM is often caused by the complex interactions between genetic and/or environmental associations^3^. T2DM is increasingly recognized as a serious public health problem, with an estimated 700 million patients worldwide by 2045, and constitutes a significant financial burden^4–6^. Currently, six classes of oral antidiabetic drugs, including metformin, glimepiride, repaglinide, pioglitazone, sitagliptin, and acarbose, are available^7^. Despite their benefits, these drugs are focused on a single compound and reported to have adverse effects. Hence, complementary and alternative medicine may be prospective options for T2DM intervention. As a supplementary alternative medicine, traditional Chinese medicine (TCM) has a rich history and has shown good results in the treatment of T2DM.

Rhizoma Coptidis is the dried rhizome of the family Ranunculaceae: *Coptis chinensis* Franch^8^. According to TCM beliefs, Rhizoma Coptidis possesses the functions of clearing heat, drying dampness, wasting thirst, and quenching fire toxins^9^. Thus, Rhizoma Coptidis is widely used by TCM physicians to prevent and/or treat diabetes^10^. Many famous formulas containing Rhizoma Coptidis, such as Ge-Gen-Qin-Lian Decoction^11^, Dahuang Huanglian Xiexin Decoction^12^, and Huanglian Decoction^13^, have also been shown to exert therapeutic effects on T2DM. Recently, some clinical trials based on Rhizoma Coptidis intervention indicated a good hypoglycaemic effect for T2DM patients and an improvement of symptoms^14,15^. Many chemical compounds of Rhizoma Coptidis, such as berberine and polyphenols, have been verified to play a notable role in regulating glycometabolism by reducing insulin resistance in liver, muscle, and adipose tissues, increasing insulin levels, improving β cell dysfunction^16^, and regulating the intestinal flora^17^. Thus, this treatment has the potential to ameliorate diabetes symptoms and suppress T2DM-induced lesions^18^. However, due to the multiple components and multiple targeting characteristics of Chinese herbal medicine, the active compounds and underlying mechanisms have not been fully elucidated.

Network pharmacology is an interdisciplinary discipline newly developed for systematic research on drugs based on artificial intelligence and big data^19^ and is characterized by its systematic nature, relevance and predictability^20^. Network pharmacology can construct a multimolecular, multitarget and multilink network model of “drug-compound-target” to understand the mechanism of Chinese herbal medicine^21^. The treatment benefits of Chinese herbal medicine are attributed to multiple components and targets that produce combined or synergistic effects^22^. Thus, the characteristics of herbs fit well with network pharmacology, making it a useful tool to quantitatively represent the mechanisms of herbs. Recently, TCM network pharmacology was proposed by Li et al.^23,24^, which integrates TCM theory with network pharmacology. The holistic view of TCM coincides with the overall systematic characteristics of network pharmacology^25^. TCM network pharmacology is a methodology that aims to reveal the biological associations among complex diseases, syndromes and herb treatments^26^. With rapid progress in bioinformatics, TCM network pharmacology has had wide applications, such as drug development, herbal ingredient target prediction, and compatibility regularity of drug pairs. Therefore, a network pharmacology approach was utilized to predict the antidiabetic mechanisms of Rhizoma Coptidis in this study.

**Figure 1:**
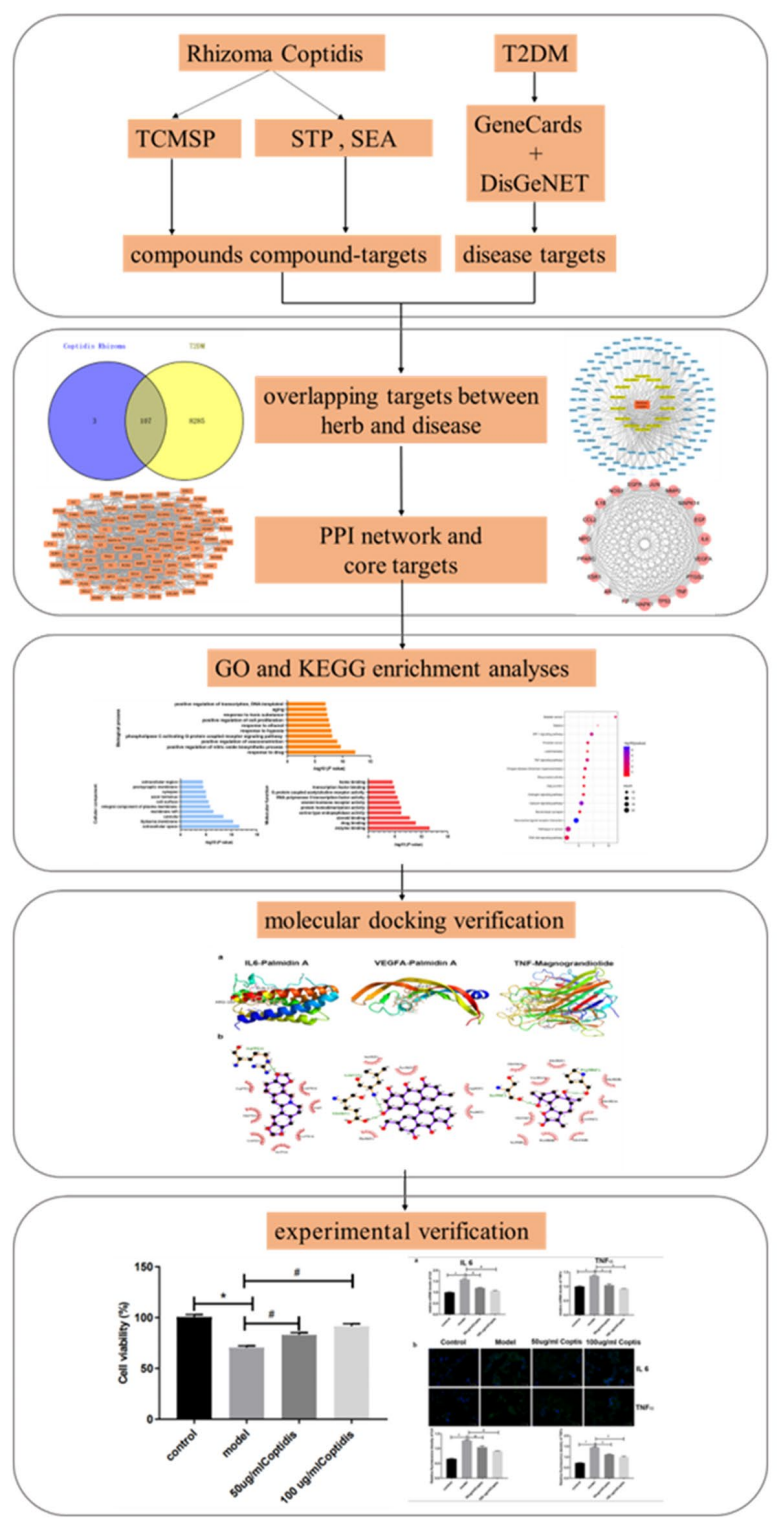
Workflow for Rhizoma Coptidis on treating T2DM.

Presently, we performed a systematic study to clarify the therapeutic effect of Rhizoma Coptidis for T2DM. The steps were as follows: the active compounds of Rhizoma Coptidis were identified by the TCMSP database, and the compound targets were predicted by the STP and SEA databases. The GeneCards and DisGeNET system was used to screen disease targets, and the overlapping targets between herbs and disease were also identified. In addition, the overlapping targets were entered into String for protein–protein interaction analysis, and core targets were screened according to topological structure. Furthermore, GO and KEGG enrichment analyses were performed using the DAVID tool to identify the functions and pathways. Finally, molecular docking technology and in vitro experiments were used to verify the results of the network pharmacology analysis. The workflow used in this study is illustrated in Fig. 1.

## Materials and methods

### Screening of active compounds of Rhizoma Coptidis

The chemical compounds of Rhizoma Coptidis were harvested using the Traditional Chinese Medicine Systems Pharmacology Database and Analysis Platform (TCMSP, https://tcmspw.com/tcmsp.php). According to literature reports and pharmacokinetic parameters^27^, the compounds with oral bioavailability (OB) ≥ 30% had good absorption and slow metabolism after oral administration. The compounds with a drug-likeness (DL) ≥ 0.18 were chemically suitable for drug development. Hence, OB ≥ 30% and DL ≥ 0.18 were employed to identify the potential active compounds in Rhizoma Coptidis^28^.

### Prediction of compound targets

Targets of the active compounds of Rhizoma Coptidis were selected through both the similarity ensemble approach (SEA) (http://sea.bkslab.org/) and Swiss target prediction (STP) (http://www.swisstargetprediction.ch/) in “Homo Sapiens” mode. The UniProt database (https//www.uniprot.org/) was used to convert target protein names into target gene names to standardize gene names^29^.

### Identification of related targets for T2DM

T2DM-related targets were searched in the GeneCards platform (https://www.genecards.org/) and DisGeNET (https://www.disgenet.org/search) with “type 2 diabetes mellitus” and “T2DM” as the key words^30^. The species was set to *Homo sapiens*. Then, these collected targets were merged, and duplicates were removed. The overlapping targets between compounds of Rhizoma Coptidis and T2DM targets were identified and visualized by VENNY 2.1 (https://bioinfogp.cnb.csic.es/tools/venny/).

### Construction of a drug–compound–target network

Based on the above analyses, the active compound and targets of Rhizoma Coptidis were matched with the disease target of T2DM to obtain the compound target. Cytoscape 3.7.2 (http://www.cytoscape.org/) was used to establish the network diagram between the active compounds of Rhizoma Coptidis and T2DM-related targets^31^.

### Construction of the protein–protein interaction (PPI) network

The overlapping targets were input into String 11.0 (Search Tool for the Retrieval of Interacting Genes/Proteins, https://string-db.org/) to obtain the interaction results among the targets with the gene type selected as Homo sapiens^32^, and the visual analysis results were obtained by Cytoscape 3.7.2.

In the topological analysis, degree reflects the number of connections between network nodes and other nodes, which is the main topological parameter used to measure the importance of a node in a network^33,34^ and determine whether a target protein is an important basis for key targets^35^. In this study, the nodes with degree values greater than twofold the median were taken as important nodes in the network, namely, “core targets”.

### Gene ontology (GO) functional annotation and Kyoto Encyclopedia of Genes and genomes (KEGG) pathway analysis

The DAVID database (https://david.ncifcrf.gov/) is an online tool for gene annotation, classification, and enrichment and pathway analysis. GO and KEGG pathway enrichment analyses were carried out using the DAVID tool. A *p* value < 0.05 and a *p* value < 0.01 were used as the thresholds for GO terms and KEGG pathways, respectively, and some top entries were chosen for visualization^36^. GO enrichment analysis mainly considers the biological process, cellular composition, and molecular function of the target, whereas KEGG enrichment analyses consider the potential biological pathways and functions associated with the target^37^.

### Molecular docking

To validate the effectiveness of the screened compounds and targets, molecular docking was carried out between the active compounds and top 3 core targets. Metformin, a commonly used clinical drug, was used as a positive control. The name, molecular weight, and 2D structure of the compounds were determined in the PubChem database (https://pubchem.ncbi.nlm.nih.gov/) as the small-molecule ligand file. The protein receptor structure file was obtained from the RCSB PDB database (http://www.rcsb.org/) in PDB format; the original ligand structure of the protein was extracted by using PyMOL 2.3 software (https://pymol.org/2/). Autodock Vina was used for molecular docking and to calculate the results. The binding degree of the receptor-ligand can be judged by the level of binding energy in the molecular docking results. If the binding energy is less than − 5 kJ/mol, it indicates that the target has certain binding activity with the compound^38^. The lower the binding energy is, the better the docking effect. After docking, ligands with the lowest binding energy were selected to visualize the ligand–protein interaction.

### In vitro experiments

#### Cell culture

Cell experiments were conducted in accordance with the guidelines and regulations of Shandong University of Traditional Chinese Medicine. Min6 cells were obtained from the Cell Bank of the Chinese Academy of Sciences. Min6 cells were cultured in DMEM (5.5 mmol/L glucose, Coring, 10-014-CVR) supplemented with 10% FBS (Gibco, 10091155) and 1% penicillin/streptomycin (Gibco, 15140163) and maintained at 37 °C in a humidified atmosphere with 5% CO_2_^39^. Rhizoma Coptidis (Voucher specimens, 1706168) was purchased from TCM Pharmacy of Affiliated Hospital of Shandong University of Traditional Chinese Medicine (Jinan, China). Rhizoma Coptidis was decocted for 30 min twice with boiling deionized water (1:10 and then 1:5, w/v). The solution was filtered and concentrated and stored at − 20°^40^. The experimental groups were divided into the control group (5.5 mmol/L glucose), high glucose-model group (25 mmol/L glucose), Rhizoma Coptidis low-dose group (25 mmol/L glucose and 50 µg/mL Rhizoma Coptidis), and Rhizoma Coptidis high-dose group (25 mmol/L glucose and 100 µg/mL Rhizoma Coptidis).

#### Cell viability assay

Cell viability was determined by CCK-8 (Tongren, ck04) assay. Min6 cells were seeded in a 96-well plate at a density of 4 × 10^3^ cells/well. After incubation for 12 h, the cells were treated with different concentrations of drug-containing medium for 48 h^41^. Then, 10 µL CCK-8 was added to each well, and the plates were incubated for 1–4 h. Finally, the absorbance was measured at a wavelength of 450 nm.

#### qRT–PCR analysis

Total mRNA was extracted using TRIzol reagent (Sparkjade, AC0101), and then gDNA was generated using a reverse transcription kit (Sparkjade, AG0304-B). Quantitative PCR was then performed using the SYBR Green PCR Kit (Takara, RR047Q)^42^. All reactions were carried out according to the manufacturer's protocol. The β-actin gene was used as an internal standard gene, and the data were quantitatively analysed by the 2^−ΔΔCt^ method. The value in the control group was set to 1.0, and all data are shown as relative mRNA expression^43^. All primers were designed and synthesized by Sangon, Ltd. The primer sequences of each gene were as follows: IL6 FORWARD, 5′-TGAACTCCTTCTCCACAAGCG-3′; IL6 REVERSE, 5′-CCGTCGAGGATGTACCGAAT-3′; TNFα FORWARD, 5′-GAGAGATGGGGGAGATAAGGAGA-3′; TNFα REVERSE, 5′-TTAGCCCTGAGGTGTCTGGTT-3′; β-actin FORWARD, 5′-CTACCTCATGAAGATCCTGACC-3′; and β-actin REVERSE, 5′-CACAGCTTCTCTTTGATGTCAC-3’.

#### Immunofluorescence staining

Min6 cells were seeded on coverslips. After formaldehyde (Beyotime, P0099) fixation, 0.1% Triton X-100 (Solarbio, T8200) penetration and 10% goat serum (Solarbio, SL034) blocking were performed. Then, the Min6 cells were incubated with antibody (IL6: Abcam, ab179570, 1:400; TNFα: Abcam, ab183218, 1:500) at 4 °C overnight. The next morning, after washing steps, the cells were incubated with goat anti-rabbit IgG/FITC antibody (Proteintech, SA00013-2, 1:200) at room temperature for 30 min and then with DAPI (Solarbio, C0060) for 5 min at room temperature^44^. Cells under coverslips were observed using a fluorescence microscope.

#### Statistical analysis

Statistical analysis was performed by using SPSS 24.0. The data were collected and are shown as the means ± standard deviations. Statistical analysis was performed by one-way ANOVA. *p* < 0.05 was considered to be significantly different^45^.

## Results

### Active compound and related targets

The active compounds of Rhizoma Coptidis were retrieved from the TCMSP database, and 48 related compounds were obtained. Fourteen related compounds were identified to have an OB ≥ 30% and a DL ≥ 0.18. Altogether, 14 compounds were considered active compounds of Rhizoma Coptidis^46^ (Table 1). A total of 110 compound targets were identified from the 14 active compounds of Rhizoma Coptidis using the STP and SEA databases to delete repetitive targets.

**Table 1:** The information of 14 active compounds of Rhizoma Coptidis.

Mol ID	Molecule name	OB (%)	DL
MOL001454	Berberine	36.86	0.78
MOL013352	Obacunone	43.29	0.77
MOL002894	Berberrubine	35.74	0.73
MOL002897	Epiberberine	43.09	0.78
MOL002903	(R)-canadine	55.37	0.77
MOL002904	Berlambine	36.68	0.82
MOL002907	Corchoroside A_qt	104.95	0.78
MOL000622	Magnograndiolide	63.71	0.19
MOL000762	Palmidin A	35.36	0.65
MOL000785	Palmatine	64.6	0.65
MOL000098	Quercetin	46.43	0.28
MOL001458	Coptisine	30.67	0.86
MOL002668	Worenine	45.83	0.87
MOL008647	Moupinamide	86.71	0.26

### Overlap analysis between Rhizoma Coptidis targets and T2DM-related targets

**Figure 2:**
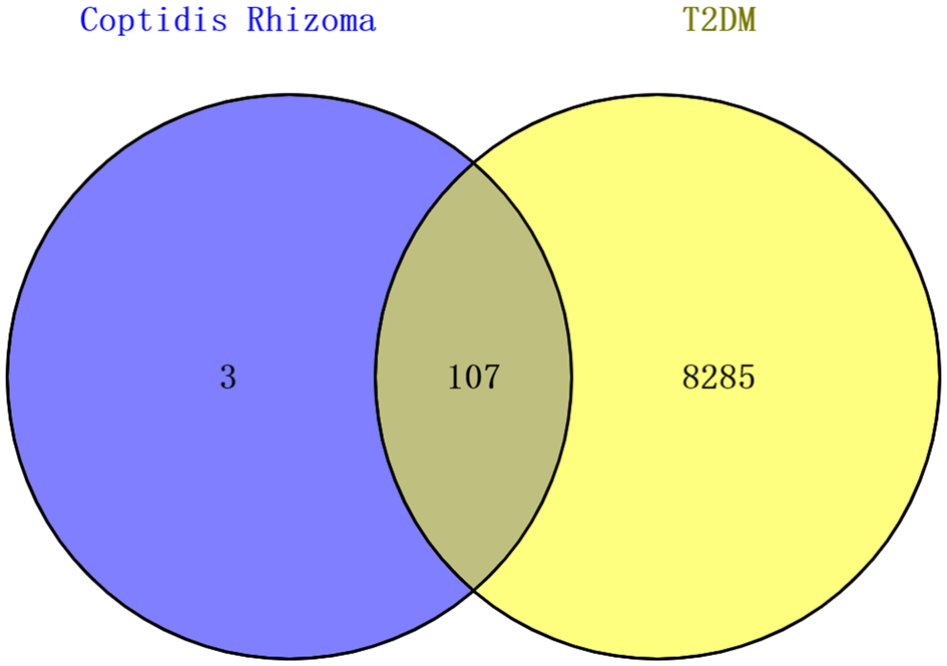
Venn diagram of the overlapping targets in Rhizoma Coptidis and T2DM.

**Figure 3:**
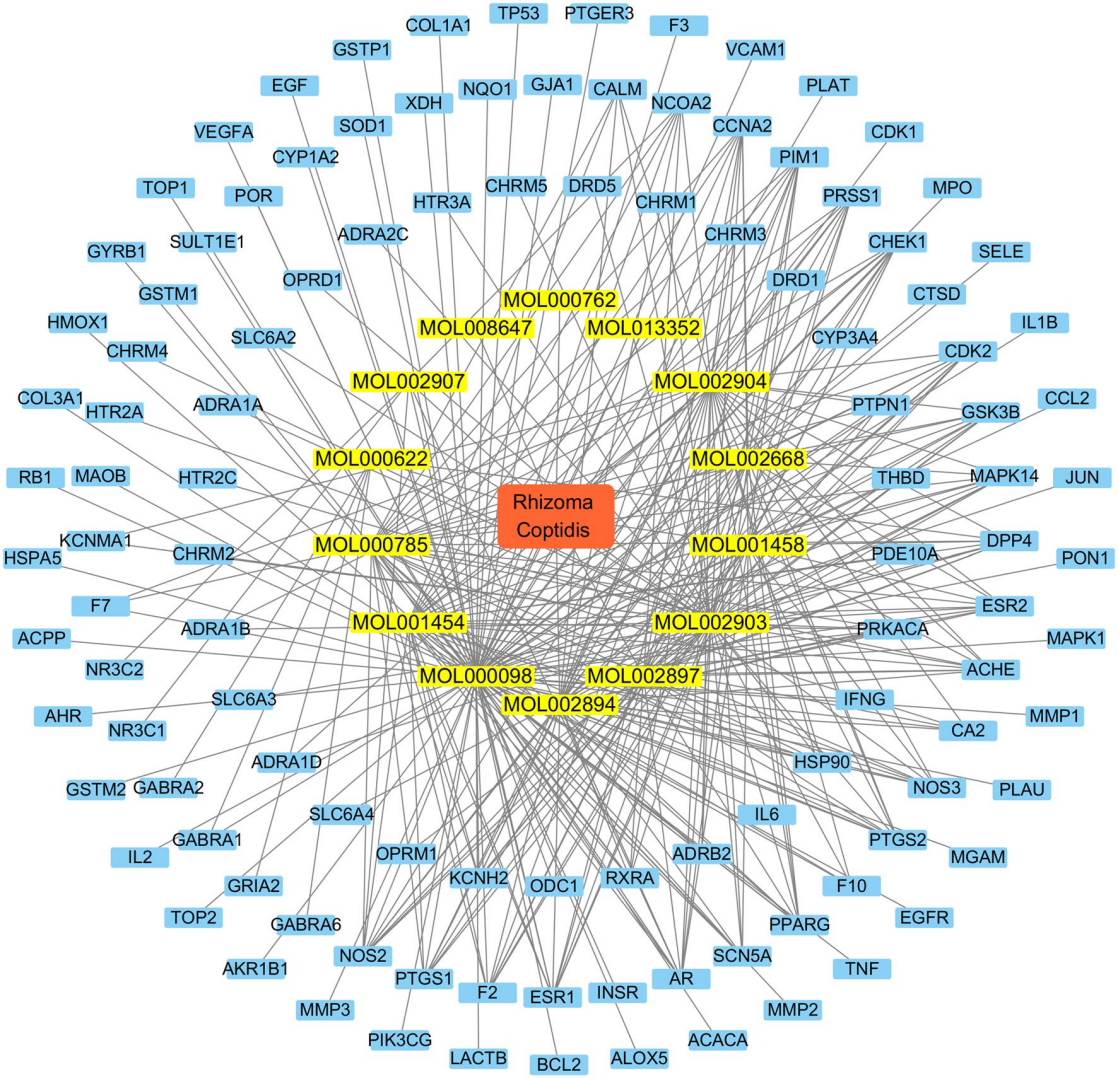
Drug-compound-target network. Red represents Rhizoma Coptidis, yellow represents compounds of Rhizoma Coptidis and blue represents targets, respectively.

A total of 8392 T2DM-related targets were acquired by screening the GeneCards and DisGeNET databases. After the intersection of 110 compound targets and 8392 T2DM-related targets, 107 overlapping targets were obtained, as shown by a Venn diagram (Fig. 2). We also constructed a drug–compound–target network using Cytoscape 3.7.2 software to further clarify the mechanism of Rhizoma Coptidis in treating T2DM (Fig. 3).

### PPI network

**Figure 4:**
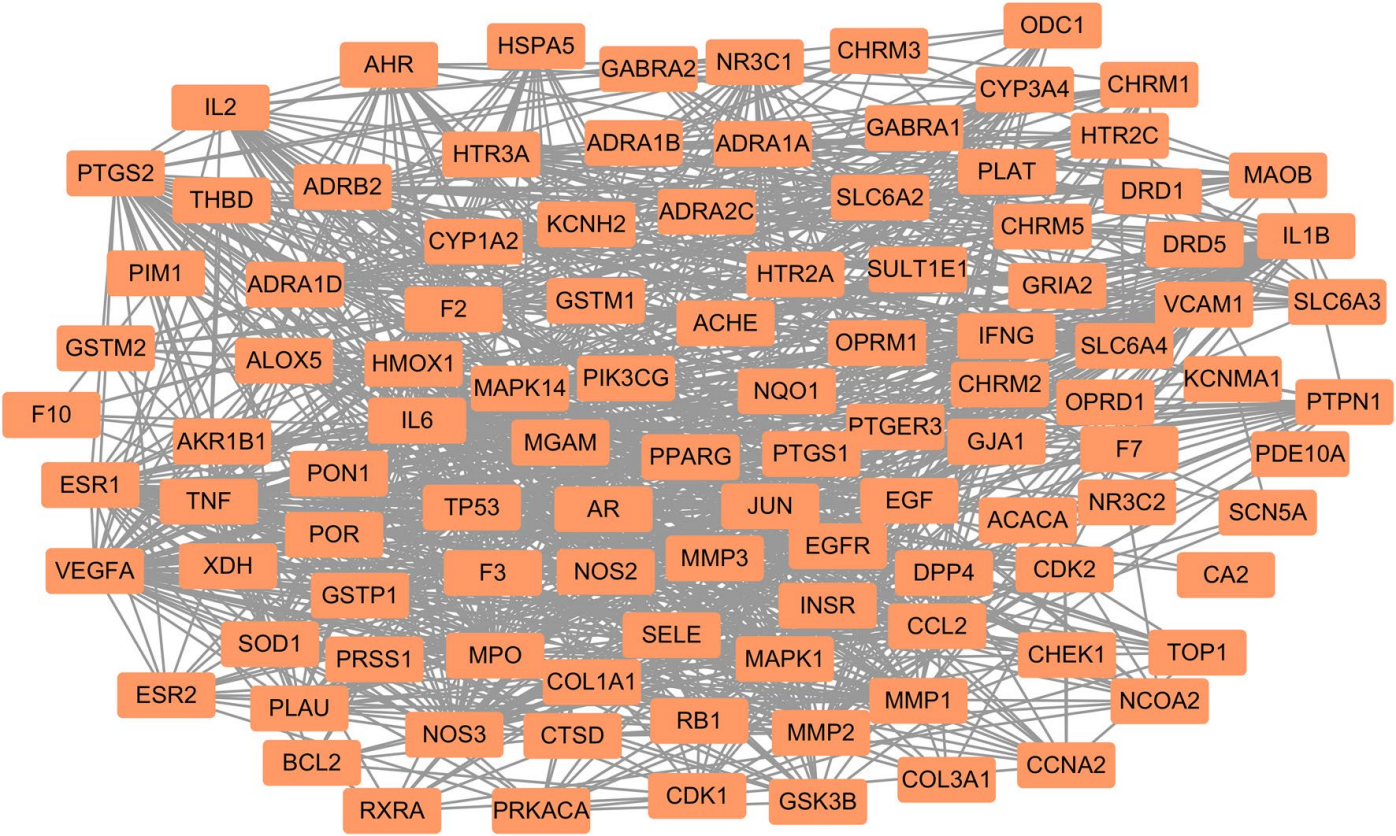
PPI network of 107 targets of Rhizoma Coptidis against T2DM. Edges represent protein–protein associations.

**Figure 5:**
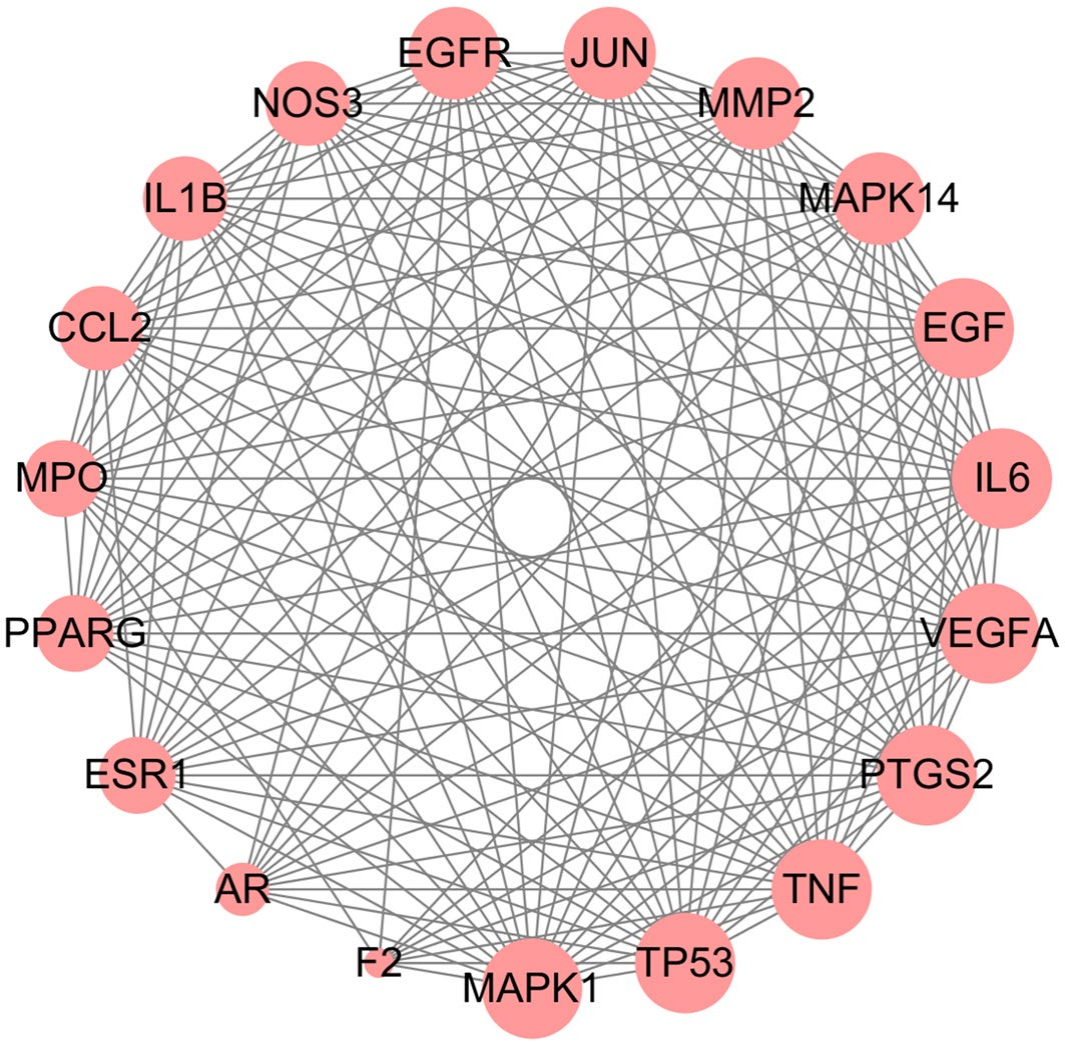
PPI network of 19 core targets of Rhizoma Coptidis against T2DM screened by degree. Nodes from small to large represent the degree value from low to high.

The PPI network of 107 overlapping targets contained 107 nodes and 1028 edges (Fig. 4). According to the twofold median degree value obtained by topological analysis, 19 core targets were further selected, including IL6, VEGFA, TNF, TP53, EGF, JUN, MAPK1, EGFR, NOS3, PTGS2, ESR1, IL1B, CCL2, PPARG, MPO, AR, MMP2, MAPK14, and F2. We constructed a network diagram of the 19 core targets, and the results are shown in Fig. 5. The target is represented by a circular node, and nodes change from small to large. Larger nodes indicate that the degree value is high and that the target is more important. Among them, IL6 had the highest degree value (degree = 116), followed by VEGFA (degree = 108) and TNF (degree = 106). Thus, these 3 targets can be recognized as the most significant core targets for Rhizoma Coptidis against T2DM and deserve subsequent study.

### GO and KEGG pathway enrichment analysis

**Figure 6:**
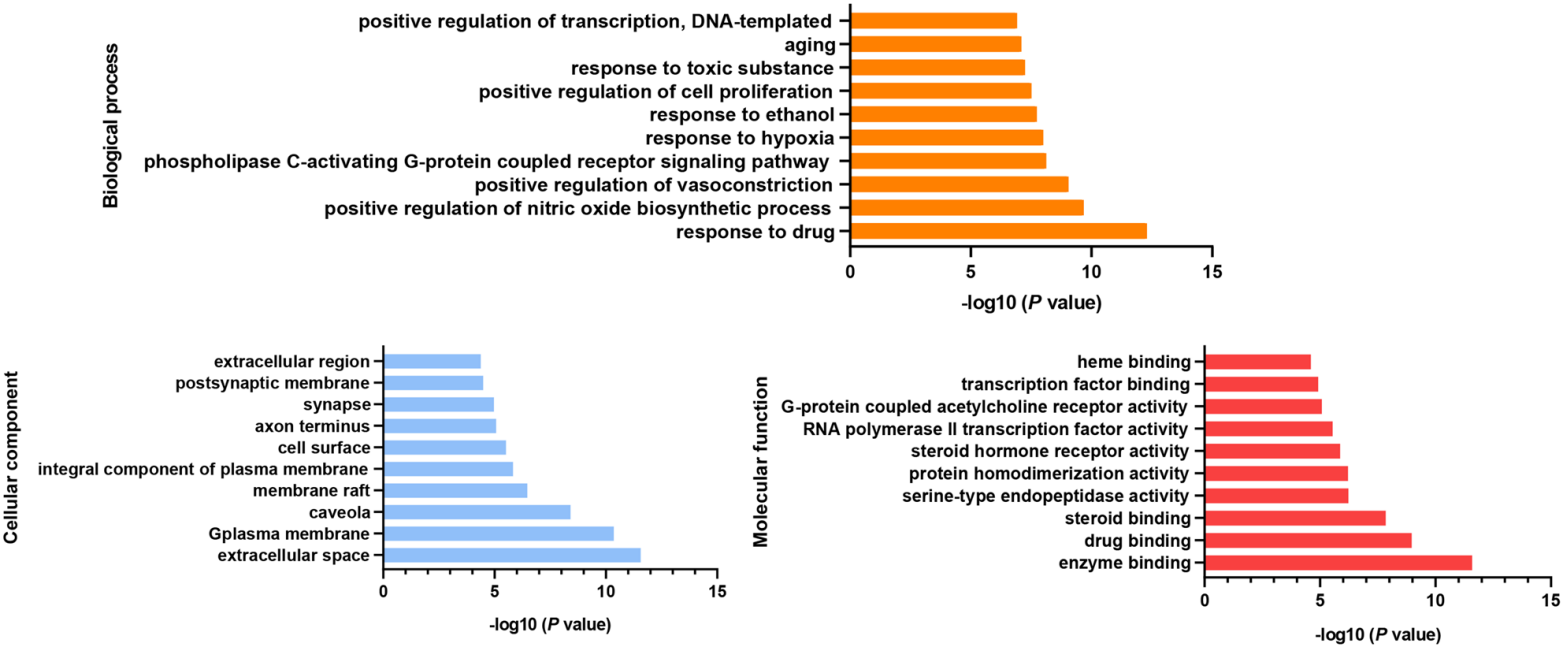
The top 10 results of GO enrichment analyses for 107 overlapping targets.

GO enrichment analysis was performed on 107 targets, and a total of 165 GO terms with *p* < 0.05 were obtained, including 110 entries of biological process (BP), 25 entries of cell composition (CC) and 30 entries of molecular function (MF). According to the *p* value, the top 5 terms in the biological process category were response to drug (GO:0042493), positive regulation of nitric oxide biosynthetic process (GO:0045429), positive regulation of vasoconstriction (GO:0045907), phospholipase C-activating G-protein coupled receptor signaling pathway (GO:0007200), and response to hypoxia (GO:0001666). These biological processes were strongly connected with the following molecular functions: enzyme binding (GO:0019899), drug binding (GO:0008144), steroid binding (GO:0005496), serine-type endopeptidase activity (GO:0004252), and protein homodimerization activity (GO:0042803). Moreover, these processes occurred mainly in the extracellular space (GO:0005615), plasma membrane (GO:0005886), caveola (GO:0005901), membrane raft (GO:0045121), and integral compounds of the plasma membrane (GO:0005887). The top 10 GO terms were visualized and are displayed in Fig. 6.

**Figure 7:**
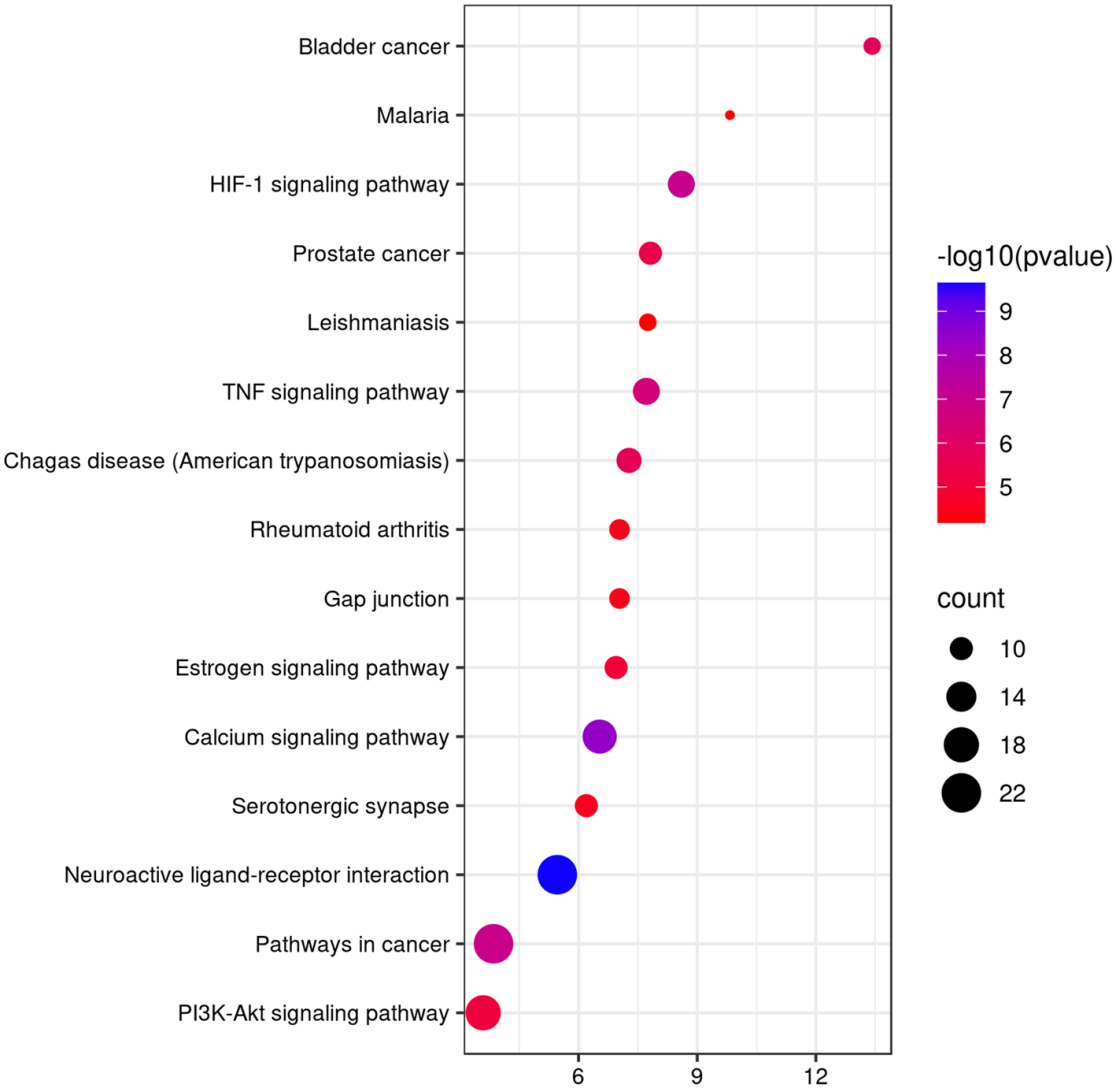
The top 15 results of KEGG pathway enrichment analyses for 107 overlapping targets. The color represents the different −log10 (p value), the size of the circle represents the counts.

The results of KEGG pathway enrichment analysis demonstrated that 107 targets were enriched in 88 signalling pathways (*p* < 0.01), mainly involving Neuroactive ligand-receptor interaction(hsa04080), Calcium signalling pathway(hsa04020), HIF-1 signalling pathway(hsa04066), Pathways in cancer(hsa05200), TNF signalling pathway(hsa04668), and PI3K/Akt signalling pathway(hsa04151). We selected the top 15 results to draw a bubble diagram (Fig. 7). The larger the bubble is, the more genes are enriched in the pathway. The redder the colour is, the smaller the *p* value and more significant the result.

### Molecular docking results

**Figure 8:**
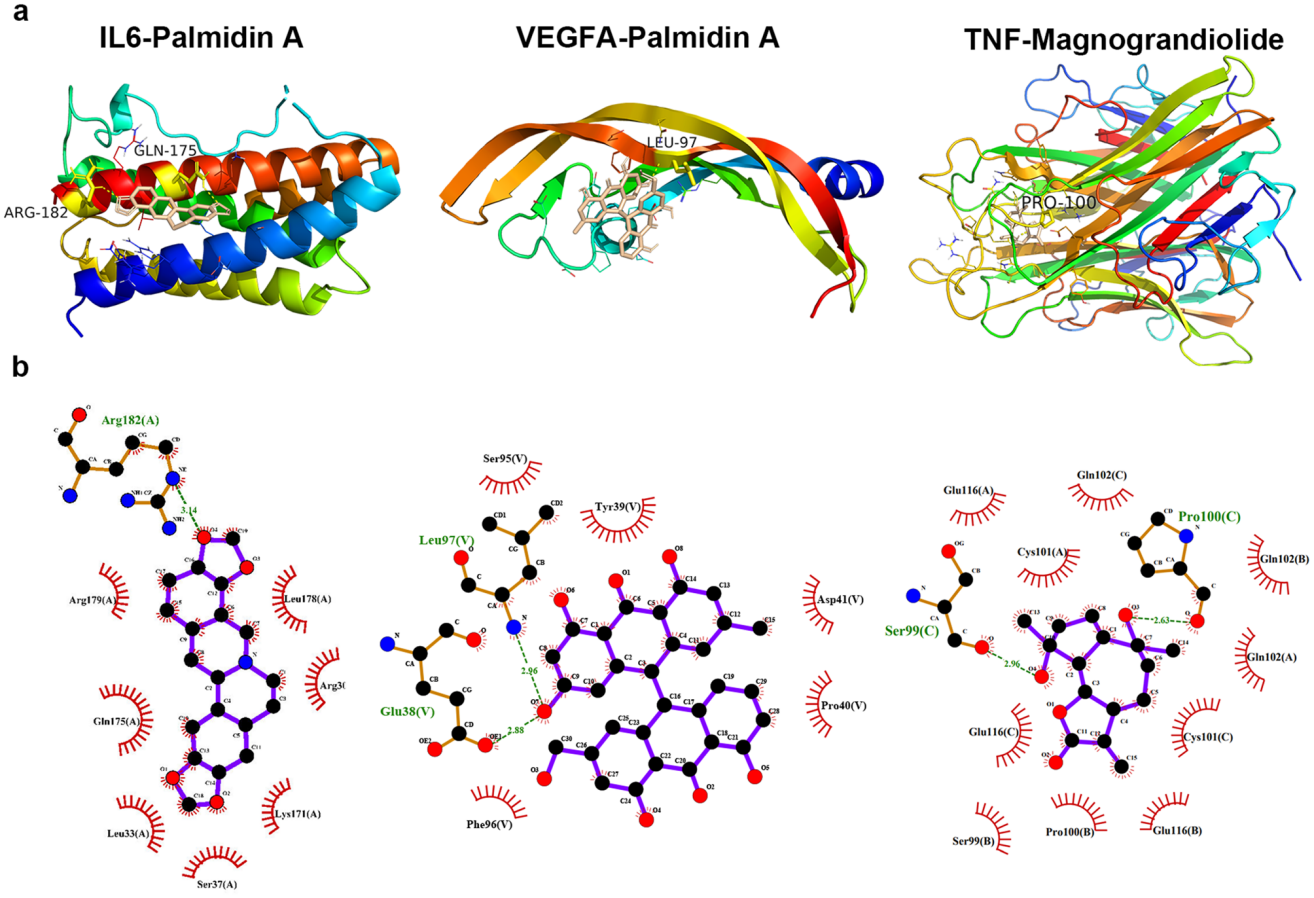
Molecular docking interaction between 3 core targets and best docked compounds from Rhizoma Coptidis. (**a**) Stereo view of interaction for IL6, VEGFA and TNF with best docked compounds from Rhizoma Coptidis. (**b**) The view of the 2-D interaction among IL6, VEGFA and TNF with above compounds.

Molecular docking was applied to analyse the binding of the top 3 targets (IL6, VEGFA and TNF) and all 14 active compounds of Rhizoma Coptidis. Their binding energy scores were all smaller than − 5 kJ/mol, and the results are listed in Table 2. In detail, the active compound with the lowest binding energy to IL6 and VEGFA was palmidin A, and the active compound with the lowest binding energy to TNF was magnograndiolide. The binding energy scores of IL6—palmidin A and VEGFA—palmidin A were − 7.6 and − 6.8 kcal/mol, respectively, whereas their binding energy scores with metformin were − 4.6 and − 3.6 kcal/mol, respectively. Additionally, the binding energy score of TNF-magnograndiolide is also lower than that of TNF-metformin (− 8.8 *vs.* − 5.7 kcal/mol). These results indicate that the screened core targets indeed stably combine with Rhizoma Coptidis. The docking results of the top 3 targets are shown in Fig. 8.

**Table 2:** Binding energy for IL6, VEGFA and TNF with compounds of Rhizoma Coptidis.

Mol ID	Molecule Name	Binding energy (kJ/mol)		
		IL6	VEGFA	TNF
Positive control	Metformin	− 4.6	− 3.6	− 5.7
MOL000098	quercetin	− 6.9	− 5.9	− 6.9
MOL000622	Magnograndiolide	− 5.4	− 5.5	− 8.8
MOL000762	Palmidin A	− 7.6	− 6.8	− 7.6
MOL000785	palmatine	− 6.4	− 5.3	− 6
MOL001454	palmatine	− 6.9	− 5.9	− 6.9
MOL001458	coptisine	− 7.6	− 6.1	− 8.5
MOL002668	Worenine	− 6.8	− 6.3	− 7.3
MOL002894	berberrubine	− 6.6	− 5.6	− 7.8
MOL002897	epiberberine	− 7.3	− 5.9	− 6.6
MOL002903	(R)-Canadine	− 6.7	− 5.7	− 6.5
MOL002904	Berlambine	− 7.1	− 5.7	− 7.8
MOL002907	Corchoroside A_qt	− 6.2	− 5.4	− 6.4
MOL008647	Moupinamide	− 6.8	− 5.3	− 6.7
MOL013352	Obacunone	− 7.5	− 6.6	− 7.5

### Effect of high glucose and Rhizoma Coptidis on cell viability

**Figure 9:**
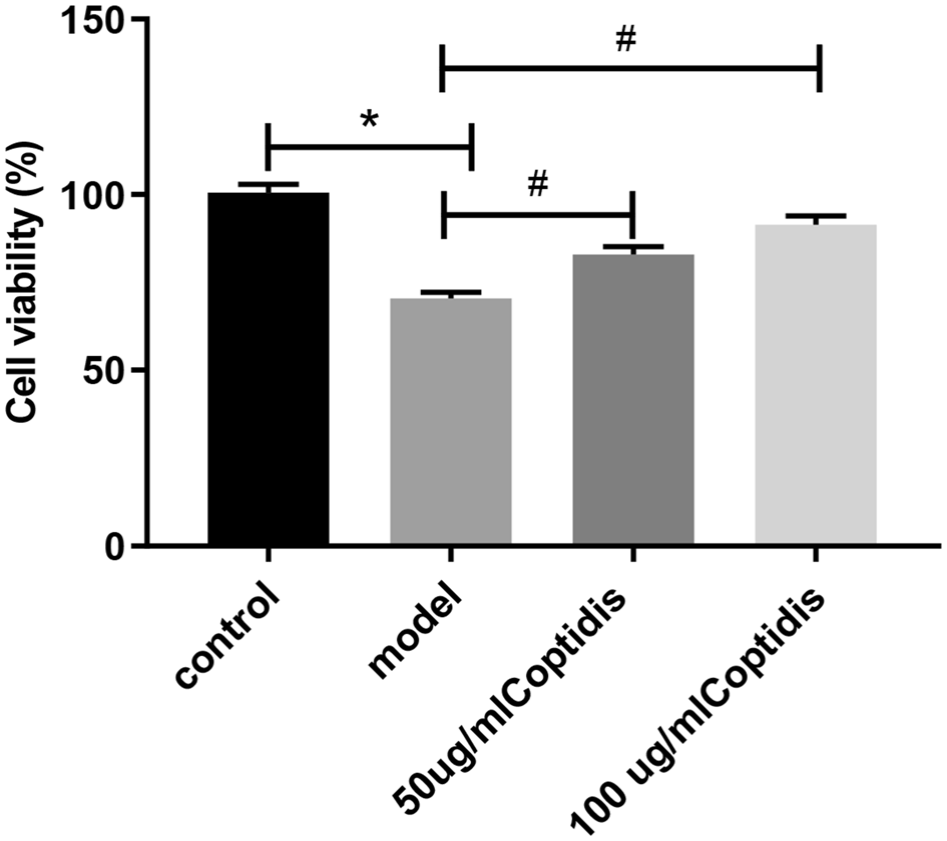
Effect of Rhizoma Coptidis on cellular viability. *p < 0.05 vs. control group; ^#^p < 0.05 vs. model group.

The effect of Rhizoma Coptidis on cell viability was detected by the CCK-8 method. As shown in Fig. 9, the cell viability of the model group decreased by 30.24% compared to that of the control group (*p* < 0.05), indicating that high glucose inhibited Min6 cell viability. Compared with the model group, different concentrations (50 µg/mL and 100 µg/mL) of Rhizoma Coptidis enhanced cell viability (increasing by 12.51% and 21.01%, respectively) (*p* < 0.05). These results suggest that Rhizoma Coptidis enhances β cell viability during high-glucose injury.

### Effects of Rhizoma Coptidis on the expression of core targets

The network pharmacology results revealed that IL6 and TNF are the core targets of Rhizoma Coptidis in the treatment of T2DM, and the TNF signalling pathway is its main intervention pathway. Additionally, the molecular docking results showed that all active compounds of Rhizoma Coptidis effectively bound to IL6 and TNF. Therefore, we selected IL6 and TNF for further verification.

**Figure 10:**
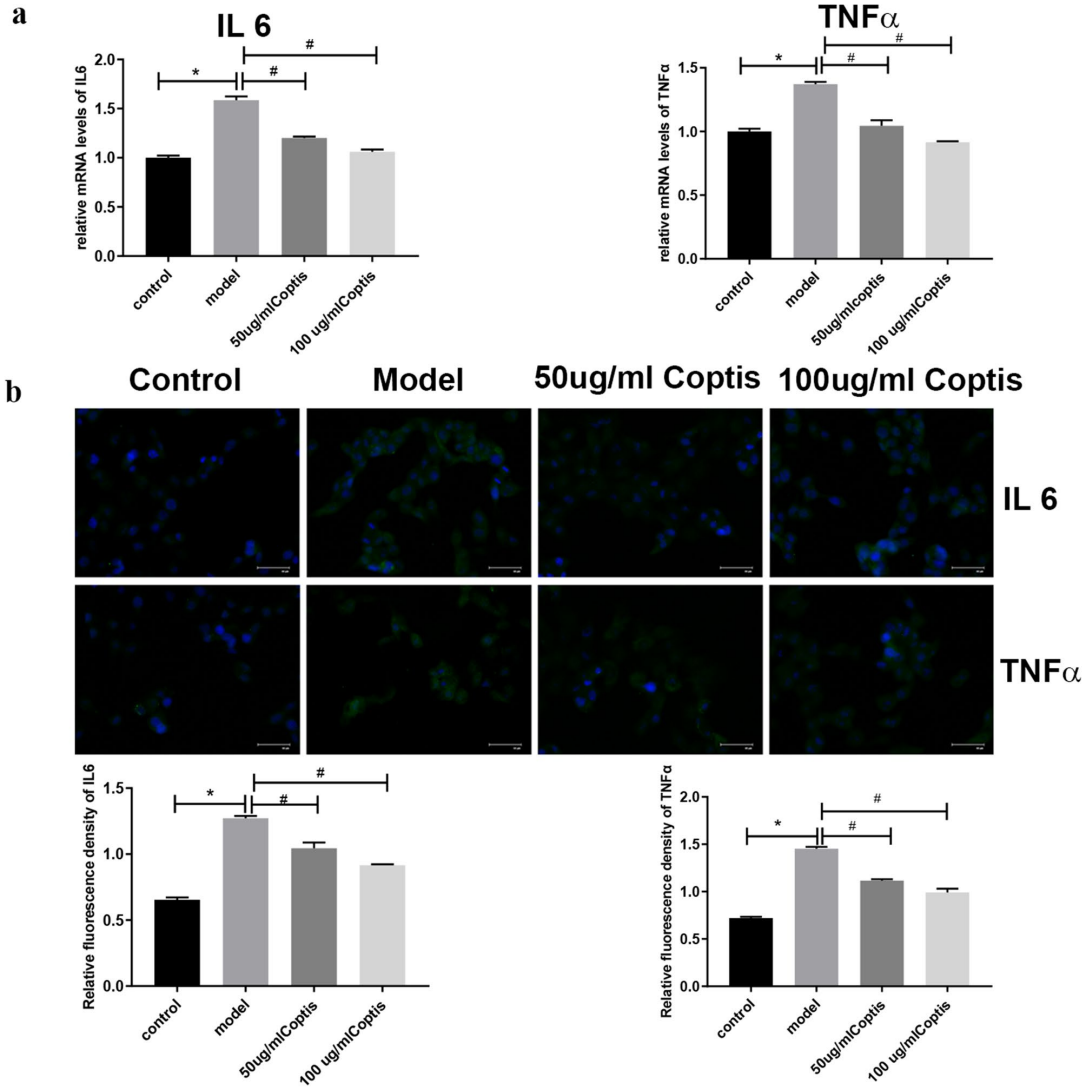
Effect of Rhizoma Coptidis on expressions of IL6, TNFα. (**a**) qRT-PCR analysis of IL6, TNFα. (**b**) Immunofluorescence staining evaluates fluorescence density of IL6, TNFα. *p < 0.05 vs. control group; ^#^p < 0.05 vs. model group.

qRT–PCR was performed to determine whether Rhizoma Coptidis regulated the mRNA expression of IL6 and TNFα in Min6 cells. The qRT–PCR results showed that IL6 and TNFα mRNA levels in the model group were increased relative to those in the control group (*p* < 0.05). Rhizoma Coptidis treatment (50 µg/mL, 100 µg/mL) helped decrease the expression levels of IL6 and TNFα compared to those in the model group (*p* < 0.05) (Fig. 10a).

Consistent with the qRT–PCR results, the immunofluorescence staining results showed that Min6 cells in the model group exhibited an increase in the fluorescence densities of IL6 and TNFα compared to those of the control group (*p* < 0.05). However, this increase was inhibited by multiple concentrations (50 µg/mL and 100 µg/mL) of Rhizoma Coptidis (*p* < 0.05) (Fig. 10b).

These results indicate that Rhizoma Coptidis regulates the expression of IL6 and TNFα in a high glucose-injured cell model. Thus, Rhizoma Coptidis inhibits the expression of IL6 and TNFα.

## Discussion

In the present study, we applied a systematic pharmacological method to predict and elucidate the potential molecular mechanisms of Rhizoma Coptidis against T2DM. Based on the screening results, the therapeutic effect of Rhizoma Coptidis on T2DM was found to be related to 14 active compounds, including berberine, Obacunone, berberrubine, epiberberine, (R)-Canadine, Berlambine, Corchoroside A_qt, Magnograndiolide, Palmidin A, palmatine, quercetin, coptisine, Worenine, and Moupinamide. The above results demonstrated the effectiveness and diversity of chemical compounds in Rhizoma Coptidis to play a pharmacological role in T2DM. The degree value of quercetin was highest, so this compound was identified as the main compound in Rhizoma Coptidis. Quercetin exerted hypoglycaemic effects potentially by inhibiting pancreatic iron deposition and pancreatic β cell ferroptosis^47^. Studies have proposed that the underlying mechanisms of quercetin action against T2DM may be by downregulating the RNA and protein levels of MAPK, TNF-α, IL-6, and IL-1B in rat pancreatic tissue^48,49^, which is consistent with the PPI network results in this study. It has also been reported that quercetin increases glucose uptake through IRS1/PI3K/AKT signalling^50^.

After prediction and screening, 107 overlapping targets were found for drugs and diseases, indicating the multiple targets of hypoglycaemia in Rhizoma Coptidis. Based on the topological analysis, we further identified 19 core targets from the 107 overlapping targets for subsequent study. Further analysis of the PPI results indicated that IL6, VEGFA and TNF were the top 3 overlapping targets, which act as the core targets of Rhizoma Coptidis in T2DM. IL6, VEGFA and TNF are primarily involved in inflammation, which suggests that Rhizoma Coptidis may have an anti-inflammatory effect. Interleukin-6 (IL6) is identified as a key mediator of inflammation, fibrosis and glucose metabolism; it is the basis of insulin resistance in patients with T2DM^51,52^. IL6 also increases the expression of insulin-degrading enzymes, which are important in glucose metabolism^53^. Vascular endothelial growth factor A (VEGFA) is a growth factor that has a vital role in angiogenesis and endothelial cell growth. Hyperglycaemia results in overexpression of VEGFA, which is a critical factor in diabetic complications such as diabetic retinopathy and diabetic nephropathy^54,55^. Similarly, as a crucial proinflammatory mediator, tumour necrosis factor (TNF) induces insulin resistance by impairing insulin signalling through serine phosphorylation^56^. In addition, TNF is mainly related to diabetic vascular dysfunction and diabetic nephropathy^57,58^. Collectively, the results of this study suggest that the core targets of Rhizoma Coptidis are of great significance in antidiabetic treatment.

Furthermore, the primary core targets (IL6, VEGFA and TNF) were verified by molecular docking and experimental studies. The molecular docking results were successful according to intermolecular interactions. Experimental studies have also confirmed that Rhizoma Coptidis can notably lessen the expression of the inflammatory cytokines IL6 and TNFα to protect islet β cells against high-glucose injury. All these results sufficiently indicate that Rhizoma Coptidis possesses good anti-inflammatory activity. Accordingly, we propose that Rhizoma Coptidis had a therapeutic effect on T2DM by regulating inflammatory cytokines.

GO enrichment analysis showed that 107 targets were enriched in biological process, cellular composition, and molecular function. Oxidative stress is the most common pathological damage process in the body and is related to the pathogenesis of multiple systems and chronic diseases such as diabetes^59^. Studies have shown that some compounds of Rhizoma Coptidis, such as berberine, have antioxidant activity, which improves insulin resistance and promotes insulin secretion by scavenging free radicals^60^, inhibiting the activation of polyol bypass and reducing the formation of terminal glycosylation products^61^. Apoptosis is the key factor in the pathophysiology of T2DM^62^. STZ-induced diabetes significantly increased the expression of apoptosis biomarkers^63^. Other studies have also confirmed the involvement of Jun, CASP3 and EGFR in islet β-cell apoptosis^64^. In addition, treatment of T2DM with Rhizoma Coptidis is also related to cellular composition and molecular function, including mitochondria, endoplasmic reticulum and cytokine activity^65^. Some studies have shown ultrastructural abnormalities in the cells of patients with T2DM, such as endoplasmic reticulum dilation and mitochondrial swelling^66^. Overall, our GO enrichment results suggest that Rhizoma Coptidis regulates various biological processes to ultimately alleviate T2DM.

In the KEGG enrichment analysis, some disease-related signalling pathways (such as Calcium signalling pathway, HIF-1 signalling pathway, TNF signalling pathway, and PI3K/Akt signalling pathway) were identified, which were closely linked to the occurrence and development of T2DM. Pulsatile insulin release from pancreatic cells is related to the frequency of Ca^2+^ oscillations. The rapid increase in intracellular Ca^2+^ triggers insulin release^67^. Ca^2+^ signalling and its dysregulation have been associated with the development of DM. Ca^2+^/cAMP signalling modulates the release of insulin from pancreatic β-cells and participates in the homeostasis of β-cells by stimulating cell proliferation and differentiation^68^. Additionally, Ca^2+^ plays a crucial role in cadmium-induced β-cell dysfunction and apoptosis^69^. The HIF-1 pathway is a well-known regulator of cellular glucose^70^. HIF may be activated during the early stage of diabetic nephropathy under hypoxia and stimulate the proliferation and aggregation of inflammatory factors in the damaged kidney^71,72^. T2DM is recognized as a chronic, low-grade inflammatory disease accompanied by elevated TNF expression. TNFα-mediated inflammation, obesity, and insulin resistance are associated with T2DM^73^. It was found that the PI3K/Akt signalling pathway was activated in high glucose‑stimulated HKC cells and was related to islet cell proliferation^74^. Insulin receptor activation through insulin binding stimulates the PI3K/AKT signalling pathway. Collectively, the findings of this study show the mode of action of the hypoglycaemic effects of Rhizoma Coptidis act through multiple targets and multiple pathways.

In conclusion, with the help of a network pharmacology strategy and molecular docking and experimental validation, this study investigated the mechanisms of action by which Rhizoma Coptidis could be used to treat T2DM. The findings suggest that the antidiabetic properties of Rhizoma Coptidis can be attributed to 14 compounds that correspond to 19 hypoglycaemic targets and are strongly related to 88 signalling pathways and many biological processes. Furthermore, molecular docking and experimentation verified the beneficial activity of Rhizoma Coptidis against 3 inflammatory factors. Overall, this study confirms that Rhizoma Coptidis might be used to treat T2DM with anti-inflammatory effects.

Network pharmacology can be used to reveal the mechanism of Chinese herbal medicine at the molecular level, and from a system perspective, it provides effective evidence and a basis to support the ameliorative effect of Rhizoma Coptidis on T2DM. However, there are also some limitations. For example, the data on various drugs, genes, and proteins are not comprehensive, and appropriate computing software has not been developed. Network pharmacology is only a reasonable predictor of the mechanism of action of Rhizoma Coptidis against T2DM. Hence, further scientific verification and more rigorous research strategies should be carried out.
